# Response actions influence the categorization of directions in auditory space

**DOI:** 10.3389/fpsyg.2015.01163

**Published:** 2015-08-07

**Authors:** Marcella C. C. Velten, Bettina E. Bläsing, Thomas Hermann, Constanze Vorwerg, Thomas Schack

**Affiliations:** ^1^Neurocognition and Action Research Group, Department of Sport Science, Faculty of Psychology and Sport Science, Bielefeld UniversityBielefeld, Germany; ^2^Center of Excellence Cognitive Interaction Technology, Bielefeld UniversityBielefeld, Germany; ^3^Ambient Intelligence Group, Center of Excellence Cognitive Interaction Technology, Bielefeld UniversityBielefeld, Germany; ^4^Institut für Sprachwissenschaft – Psycholinguistik and Center for the Study of Language and Society, University of BernBern, Switzerland

**Keywords:** auditory space, response actions, spatial categorization, spatial directions, turning movements

## Abstract

Spatial region concepts such as “front,” “back,” “left,” and “right” reflect our typical interaction with space, and the corresponding surrounding regions have different statuses in memory. We examined the representation of spatial directions in the auditory space, specifically in how far natural response actions, such as orientation movements toward a sound source, would affect the categorization of egocentric auditory space. While standing in the middle of a circle with 16 loudspeakers, participants were presented acoustic stimuli coming from the loudspeakers in randomized order, and verbally described their directions by using the concept labels “front,” “back,” “left,” “right,” “front-right,” “front-left,” “back-right,” and “back-left.” Response actions varied in three blocked conditions: (1) facing front, (2) turning the head and upper body to face the stimulus, and (3) turning the head and upper body plus pointing with the hand and outstretched arm toward the stimulus. In addition to a protocol of the verbal utterances, motion capture and video recording generated a detailed corpus for subsequent analysis of the participants’ behavior. Chi-square tests revealed an effect of response condition for directions within the left and right sides. We conclude that movement-based response actions influence the representation of auditory space, especially within the sides’ regions. Moreover, the representation of auditory space favors the front and the back regions in terms of resolution, which is possibly related to the physiological characteristics of the human auditory system, as well as to the ecological requirements of action control in the different regions.

## Introduction

Spatial concepts are commonly used to respond to questions about the locations of objects, in instructions for navigation, in narratives and reports (Vorwerg, 2001). In many communicative situations, the speaker usually constructs a mental map of the environment and translates it into spatial concepts that can be verbalized, while the listeners have to transfer the speaker’s spatial concepts into their own mental maps. The same is true for communicating the locations of sounds (representing sound source objects). In contrast to visual object localization, sound objects can be perceived outside the visual field. The processes of building mental maps and translating information are associated to spatial perception and representation. Perception relates to the processing of stimuli registered by the sensory receptors (e.g., Mark, 1993; Rookes and Willson, 2006), and representation refers to the system of symbols that have the same form as the represented object, allowing a person to make inferences about this object through processing of the symbols (Gallistel, 2001). This system of symbols comprises, for example, a set of conventions about how to describe an object (Winston, 1984). Applied to the context of auditory spatial cognition, perception can be related to the processing, recognition, and interpretation of sounds registered by the auditory sensory receptors, and representation comprises conventions for categorizing, describing, or interpreting the perceived auditory spatial information.

To evaluate the general spatial perception, a range of studies has investigated the precision in localizing the directions of objects, (e.g., Lewald and Ehrenstein, 1996; Arthur et al., 2008; Philbeck et al., 2008) and sounds (e.g., Makous and Middlebrooks, 1990; Blauert, 1997; Lewald, 2002). These studies have revealed that stimuli in the frontal region are perceived and indicated more accurately, and accuracy decreases with the eccentricity of the stimulus in relation to the viewer’s or listener’s midline.

To assess the conceptual representation and communication of the surrounding directions, most recent studies employed visual stimuli (e.g., Franklin et al., 1995; Logan, 1995; Gibson and Davis, 2011). Franklin et al. (1995) instructed their participants to describe the directions of object locations in an egocentric frame of reference using spatial concepts such as “front,” “ahead,” “back,” “rear,” and “left.” Participants’ descriptions of the regions front, back, left, and right varied in the use of (secondary direction) qualifiers, with directions in the front area being described with the greatest discriminative detail. In addition, “front” was used less frequently in single-direction descriptions than the other three direction categories. The authors argue that these findings point toward different degrees of resolution in conceptual representation for the different regions, reflecting one’s typical interactions with these regions, and in part stemming from perceptual differences. Studies on the time it takes to determine object directions in surrounding regions also confirmed the precedence of the frontal region over the others (e.g., Franklin and Tversky, 1990; Bryant et al., 1992; de Vega, 1994), with symmetry between left and right (Franklin and Tversky, 1990; Bryant et al., 1992; Franklin et al., 1995). The primacy of the front region in terms of accuracy in perception and resolution in representation is frequently explained by the fact that visual stimulation, locomotion, and manipulation generally occur in a person’s front (e.g., Logan, 1995; Tversky, 2003). Because the viewer’s gaze is usually directed to the front, the spatial attention is typically focused in this region, which also explains its primacy in terms of representation. However, the conceptual representation of the surrounding auditory space, as well as its relation and interaction with visual space, have been scarcely investigated so far.

In a recent study on the categorization of auditory space, Campos et al. (2013) corroborated the perspective of the front as the most privileged region, but adding that the categorization of the rear region might be very distinctive in comparison to the sides. While standing in a steady position, participants used similarity judgments (in the first experiment) and verbal labels (in the second experiment) to categorize egocentric directions of sound sources. In both cases, the spatial resolution of the front and back regions was higher than the side regions. The authors reasoned that these results were based on both the physiological features of the human auditory system and the ecological requirements of action control. Sounds coming from the sides typically evoke reorientation movements; front and back, in contrast, instigate no direct orienting reaction, and have therefore a special status in egocentric space. These results and their interpretation bring up the question in how far natural response actions, such as orientation movements toward the sound source, would affect the categorization of egocentric auditory space.

Turning the head toward the direction of a sound is a natural behavior that has the functional purpose of bringing the sound source into the visual field for further processing by this sense (e.g., Schnupp et al., 2011). Moreover, such turning movements of the head facilitate the localization of sound directions (e.g., Wallach, 1940), although they are not completely necessary in such tasks (see Aytekin et al., 2008, for an extended discussion). In communicative situations, speakers typically point with the arm and hand toward relevant objects or sounds, for example, while indicating directions. Because of their ecological values, head turning and arm pointing are often utilized to investigate the accuracy of participants on retrieving sound directions (e.g., Pinek and Brouchon, 1992; Haber et al., 1993a; Carlile et al., 1997).

Specifically comparing response conditions, Haber et al. (1993a) found that pointing methods involving body parts (e.g., head turning as if “pointing with the nose,” or pointing with index finger) or extensions of body parts (e.g., a cane or a stick) resulted in best accuracy of pointing to auditory directions in blind adults. In another study comparing response conditions (Pinek and Brouchon, 1992), sighted participants generally undershot auditory targets with head turning in comparison to arm pointing. In the same study, participants with right parietal damage also produced dissociated manual pointing and head turning deficits: head turning deficits tended to appear peripherally in both auditory hemifields, while manual pointing deficits tended to appear unilaterally in the left hemifield.

The differences in performance in auditory localization tasks found in studies that employed arm pointing and head turning are likely to be due to the distinct motor responses rather than to differences in perception (e.g., Haber et al., 1993a). If the head is free to turn toward the sound source in both situations, this head movement facilitates sound localization by placing the stimulus in a plane perpendicular to the interaural axis, where static localization can be optimized (Makous and Middlebrooks, 1990; Middlebrooks and Green, 1991). Hence, the differences in localization accuracy can be explained based on the different levels of sensorimotor organization involved in head turning and arm pointing, that is, an axial head-centered level and a segmental visuomanual level, respectively (Pinek and Brouchon, 1992). Arm pointing involves visuomanual coordination, which includes the integration of proprioceptive body and segment position information, as well as the relation between the target position, the body and the hand (Pinek and Brouchon, 1992). Boyer et al. (2013) also addressed the question of modularity in motor control when considering the coordination of head orientation and hand movements. The authors studied the spatial and temporal organization of participants’ hand pointing movements toward unseen auditory targets under four conditions, which included short and long auditory stimuli, and congruent and incongruent continuous auditory feedback. The long duration condition produced higher accuracy and earlier automatic head orientation toward the sound source, and the authors suggested that the heading direction toward auditory events and the motor command for reaching share the same body-centered reference frame. On the other hand, pointing with the head commonly produces errors associated with free movement of the eyes, so that participants typically visually “capture” the target position without completing the turn of the head, consequently undershooting the actual target position in terms of response, but not necessarily in accuracy of perception (e.g., Pinek and Brouchon, 1992; Carlile et al., 1997).

However, it is also possible that different types of movements toward a sound source affect the perception of its location, and not only its reproduction. This supposition is in line with the *common coding approach* to perception and action (e.g., Prinz, 1990, 1997; Gallese et al., 1999; Hommel and Müsseler, 2006), and with the *Theory of Event Coding* (Hommel et al., 2001), according to which perceived events and planned actions share a common representational domain, and therefore mutually affect each other. These theoretical frameworks diverge from earlier approaches, which stated that perception might lead to action, but is independent of it. In the contexts of the present study, the common coding account suggests that head turning and arm pointing responses used to retrieve the locations of sound sources might lead to differences in the perception (and consequently in the representation) of those locations, even though the auditory perceptual cues of turning the head might remain the same for both responses. Head turning in particular represents an action that is under natural conditions so closely linked to auditive perception that it can be regarded as part of the process of actively *perceiving*, or creating an (acoustic) event (see Hommel, 2009).

Taking into account the considerations regarding the effect of different response actions on the perception of sound sources’ location, and the ecological values of such response actions for communicating those locations, we suppose that the response action used to localize sound sources should affect the verbal categorization of auditory space. To investigate this issue, we examined the distribution of the spatial labels used to describe the egocentric directions of sound sources under three response conditions, namely: (1) responding verbally while facing front; (2) turning the head and upper body to face the stimulus, then responding verbally while facing the perceived sound source; and (3) turning the head and upper body plus pointing with the hand and outstretched arm toward the stimulus, then responding verbally while facing and pointing toward the perceived sound source. [Note that a part of the results of the facing front condition has already been published in an earlier study (Campos et al., 2013), and will be reproduced here for comparison between the conditions.] Between and within these conditions, we compared the regions associated to the given direction labels.

The first hypothesis is that the facing-front condition would produce a more generalized labeling of the directions than both conditions that allowed turning the head toward the sound source. It was expected that the participants would frequently apply the simple labels (front, back, left, right) to directions other than the cardinal ones, instead of consistently using the combined labels for intermediate directions. This assumption is based mainly on the richer (auditory and proprioceptive) cues provided by turning the head into the direction of the sound, in comparison to keeping the head straight ahead.

The second hypotheses is that turning the head plus pointing with the arm would produce more detailed verbal responses than turning the head without arm pointing. This supposition has two bases. Firstly, when communicating directions, speakers typically point with the arm toward the intended direction (rather than only looking at it), and therefore this action might lead to a more detailed semantic representation than head turning on its own, which is more closely linked to the perceptual than to a communicative process. Secondly, the arm pointing movement provides the person with a stronger proprioceptive and kinesthetic feedback, which might result in a more detailed embodied representation and thereby help to categorize the pointed location in relation to the own body.

The third hypothesis is that differences between the conditions would occur prominently in the side regions, in which spatial resolution is generally rather low, whereas the front and back regions, that have been found to have better spatial resolution, would be categorized more consistently across the conditions.

Given the findings of better performances in perceiving sounds located nearby the listeners’ midline (e.g., Makous and Middlebrooks, 1990; Blauert, 1997; Lewald, 2002), and the fact that turning movements toward the sound sources facilitate their localization (e.g., Makous and Middlebrooks, 1990; Middlebrooks and Green, 1991; Aytekin et al., 2008), we emphasize that our study focuses on the representation of sound directions, which is an often neglected issue in auditory spatial cognition, rather than the precision in localizing those sound sources. For this purpose, we have set the apparatus so, that the distances between the loudspeakers were larger than the minimum audible angle commonly reported in the literature (e.g., Mills, 1958; Middlebrooks and Green, 1991; Litovsky, 1997). Furthermore, we chose the finger snap stimulus, which is a typically familiar sound, to assure that the task of localizing the sound was easy to accomplish. We focused on the verbal categorization responses and on the question of how this categorization is influenced by typical actions when sounds’ directions have to be retrieved in everyday life. The two response actions chosen here are most closely linked to the perception of sounds, one (head turning) being part of the natural process of active auditive perception, the other one (pointing) being a most typical human gesture for indicating spatial directions in a communicative context, and therefore closely linked to (pre-) verbal concepts of space (see Tomasello, 2010). The knowledge of the concepts used for describing directions of sounds is useful for navigation guided by auditory events or verbal cues, and especially relevant for blind individuals, who need to rely on audition and descriptions of scenes more often than sighted do. Hence, besides extending the already existing findings on auditory spatial representation in sighted individuals, the present study might also be used as reference for studies with blind individuals, regarding their perception and representation of space, in comparison to sighted.

## Materials and Methods

### Participants

Twenty-four students from Bielefeld University (16 female; mean age: 24.8 years, range: 19–39, 20 right-handed), native speakers of German, took part in the study. All participants gave written consent prior to the experiment, and reported being free of any known hearing deficiencies and/or neurological impairments. This study was conducted in accordance with the ethical standards of the 1964 Declaration of Helsinki.

### Apparatus and Sound Stimuli

**FIGURE 1:**
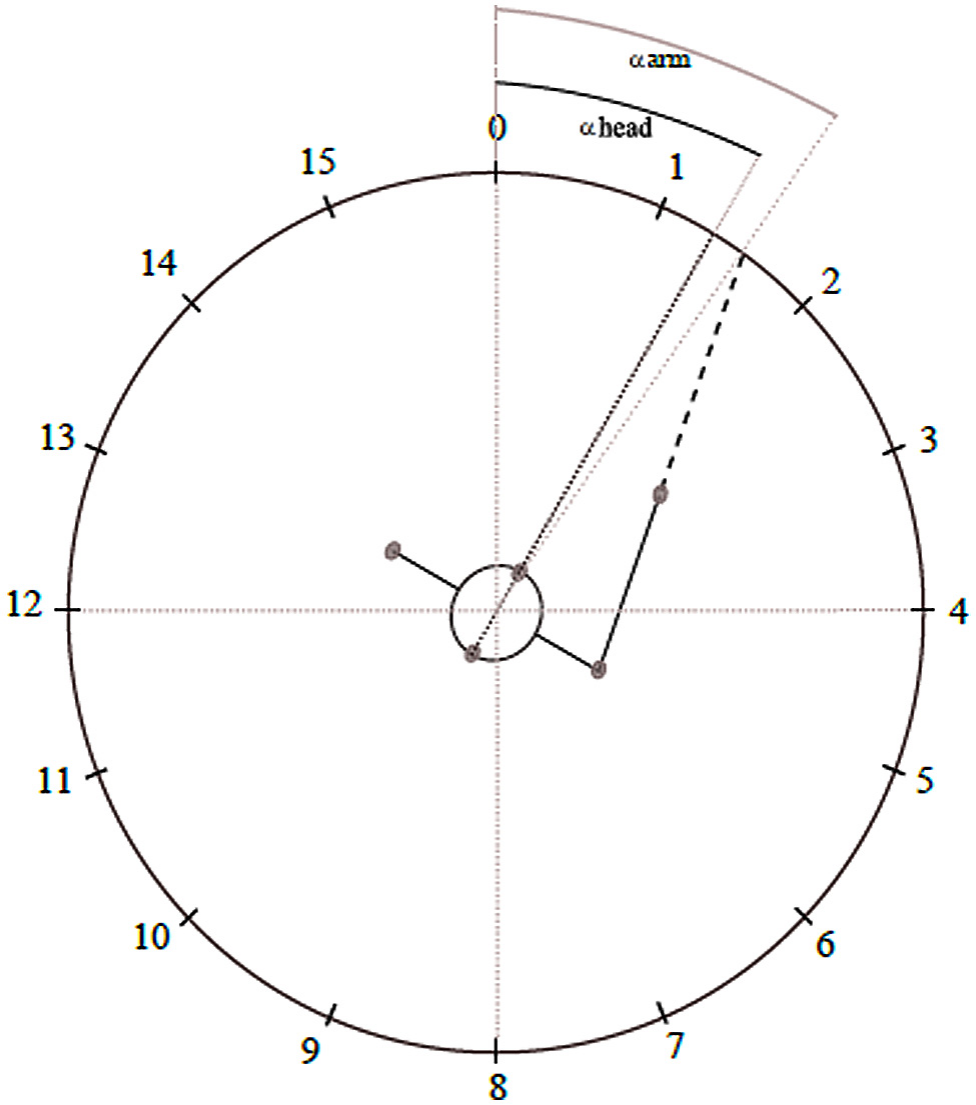
**Test room and measurement of the response actions**. The numbers represent the positions of the loudspeakers (LSs) in relation to the participant, whose initial position was in the middle of the test room, facing LS 0. The clockwise following LS positions were placed equidistantly around the ring (22.5° distance between the middle of two subsequent LSs). The gray small circles represent the reflective markers on the participant’s shoulders, head and index finger. In this example, we illustrate a movement response to a stimulus coming from LS 1. The head turning response in degrees (αhead) refers to the angle formed by the projection of the vector formed by the markers at the frontal bone and above the occipital protuberance, in relation to LS 0; likewise, the arm pointing response (αarm) refers to the angle formed by the projection of the vector formed by the markers on the shoulder and index finger, also in relation to LS 0 (0°). In this case, αhead is 30° and αarm is 34°. Therefore, as the actual position of LS 1 is at 22.5°, the error values are -7.5° and -11.5° respectively for head turning and arm pointing.

Experiments were conducted in a room which consisted of a ring (2 m outer radius) hanging from the ceiling (2.0 m above ground), with 16 Genelec 8020 loudspeakers (LSs) attached to the ring and positioned at intervals of 22.5° pointing toward the center, where the listener’s head was located. The inner radius, i.e., the actual distance between the speaker surface and the center, was 1.68 m. For further reference, each LS direction is labeled with a number from 0 to 15 (**Figure 1**). Six ‘Bonita’ cameras equidistantly attached to the ring recorded participants’ three-dimensional movements in space at 50 Hz using an optical motion capture system (Vicon Motion Systems, Oxford, UK). For this recording, 16 reflective markers (14 mm in diameter) were placed on the participant’s head, arms and upper body [four markers around the head (front middle of frontal bone, about left inferior temporal line, about right middle of parietal bone, about 3 cm above the occipital protuberance), one on each shoulder (coracoid process), two on each elbow (medial and lateral epicondyles), two on each hand (styloid processes of Radius and Ulna), and one on the middle phalange of each index finger]. Additionally, a VHS camera (Sony) positioned exactly below LS 0, i.e., in front of the subject, recorded the experiments for documentation.

A black curtain hanging from the ceiling down to the floor covered the LSs; therefore, the participants could not see the LSs, but could see the environment and their own body. Participants were not blindfolded in order to keep the experimental condition as natural as possible. The fabric of the curtain only negligibly disturbed the perceived sound, so there was practically no effect on the sound distribution.

A single spatially-fixed stimulus consisted of a series of three finger snap sounds with an inter-snap time difference of 500 ms, provided by Freesound.org (http://www.freesound.org/samplesViewSingle.php?id=11869). This stimulus was chosen because of its high localization information. The sample length was 25 ms, from the snap transient to the end of the sample. The energy was roughly concentrated around a 5 ms time segment. The transient snap sound was spectrally very broadband and exhibited a maximum at 2753 Hz (-20 dB) corresponding to a wavelength of 16.5 samples at the used sample rate of 44100 Hz. The stimuli were resynthesized and spatialized using the programming language SuperCollider and processed by the Fireface card from RME. The intensity was not measured in terms of sound pressure level (which would be difficult for such sparse signals), but instead was adjusted manually to be well-audible for the participants.

### Procedure

Participants were tested individually. While standing in the middle of the circular room, participants were asked to categorize the direction of the sound source using exclusively one of the 12 following labels: front, back, left, right, and combinations of these (e.g., front-right, right-front, etc). Participants actually used the correspondent terms in German *vorne*, *hinten*, *rechts*, *links*, and combinations of these. For further processing, we divided the labels into simple (front, back, left, and right) and combined labels, with the latter being defined as front-back/sides (FB/S; front-right, front-left, back-right, back-left) and sides/front-back (S/FB; right-front, right-back, left-front, and left-back).

Response conditions were blocked, and the following blocks were presented in random order for each participant:

Facing-front condition (FFc): In each trial, the sound stimulus was played by one of the 16 LSs, after the experimenter triggered the playback. The participant verbally defined the direction of the sound in relation to his or her own position using one of the labels described above, while maintaining his/her head and trunk facing front. Once the verbal response was given and registered by the experimenter, and the participant indicated to be ready, the experimenter triggered the playback of the next trial, with the stimulus being played by another LS. Thus, there was no fixed inter-stimulus time.

Head condition (Hc): The procedure was the same as in FFc, but as soon as the stimulus started, the participant turned his or her head and trunk to face the perceived direction of the sound source, and then verbally defined this direction using the same labels as in FFc. Participants were asked to always keep the feet oriented toward the forward direction for ensuring that the initial position (facing front) would remain the same throughout this condition, and that initial reference of front would not change within the trials. After responding, the participant turned back to the initial position and the next trial began.

Head-arm-pointing condition (HAPc): The same procedure as in Hc was applied, but, before verbally defining the direction of the sound, the participant additionally pointed with his/her closest hand and outstretched arm toward the perceived stimulus source location (i.e., with the left arm for stimuli to the left and with the right arm for stimuli to the right). After responding, the participant turned back to the initial position.

Each condition comprised five blocks. In each block, all 16 LSs were presented once in randomized order. Since the blocks were consecutive with no interval between, participants were not aware of these divisions. After each condition, participants had a break of 2 min. Each of the 24 participants completed 240 trials altogether. In each condition, 1920 trials were completed in total, and each LS was presented 120 times.

### Analysis

The first step in the analysis consisted in excluding non-valid trials from the data. Trials including errors due to technical failure (e.g., when a LS did not play the stimulus or when the participant did not respond properly) were excluded from the analysis (this applied to two trials in FFc, six in Hc and three in HAPc). Additionally, we extracted from the data all errors caused by front-back confusion (FBC), which allude to the mislocation of an acoustic stimulus when a sound located in the front is perceived as located in the rear (and vice-versa), in mirror symmetry in relation to the interaural axis (e.g., Middlebrooks and Green, 1991; Schnupp et al., 2011). Although this type of error clearly reflects inaccuracy in perception, they are qualitatively very different from “common” errors, where the perceived location is within about 20° of the actual target location (e.g., Carlile et al., 1997). Therefore, these errors are usually extracted from the responses and analyzed separately in studies of sound localization, in order to avoid distortion or overestimations of the mean or variation by outliers. As has been done in previous studies (e.g., Makous and Middlebrooks, 1990; Carlile et al., 1997), we defined that FBC was any erroneous estimate that crossed the lateral axis. For instance, when LS 2, to the front of the absolute right (LS 4), was labeled as BR. FBCs occurred in 47 trials (2.46%) in FFc, in 33 trials (1.72%) in Hc, and in 30 trials (1.56%) in HAPc. The removal of errors resulted in remaining 1871, 1881, and 1887 valid responses for FFc, Hc, and HAPc, respectively.

After extracting the non-valid responses, we analyzed the general use of the verbal labels across the LS directions and response conditions. We tested whether participants would discriminate in their use of combined labels between primary directions (i.e., for example, whether participants would use the label “front-right” more often for directions closer to the front, and “right-front” for directions closer to the right). Similar as in Vorwerg (2009), this hypothesis did not bear out, and a later analysis revealed that the FB/S labels were systematically more often used (74.54% of the responses with combined labels) than the S/FB labels, independently of the direction of the stimulus (see Vorwerg, 2009, for results on within-discourse consistency as a factor of direction order). Therefore, we reduced the combined labels regardless of the order by pooling corresponding FB/S and S/FB labels (e.g., we merged “front-right” and “right-front” into one category). This resulted in eight labels describing the 16 directions, namely: front (F), front-right (FR), right (R), back-right (BR), back (B), back-left (BL), left (L), and front-left (FL).

For each response condition, we computed the frequency of responses of each label for each LS in absolute values (i.e., assigning a value of 1 for a response and 0 for no response). We then averaged the number of responses for each participant and LS, so that all their responses to each LS sum 1. We did this in order to equalize the contributions of each participant, because of the excluded trials due to front-back or verbal confusions. Next, we tested the most frequent response to all other given responses pairwise. Because the response data was not normally distributed, we compared them using Wilcoxon signed rank test, with Bonferroni adjusted *P-*value of 0.05/k, where k is the number of comparisons for each LS. In this case, because we only compared the most frequent response to the other given responses, k is coincident with the degrees of freedom (df). Therefore, we have for four given responses (df = 3) a *P-*value of 0.05/3 = 0.017; for three given responses (df = 2), a *P-*value of 0.05/2 = 0.025; and for two given responses, a non-modified *P-*value of 0.05.

Due to the categorical nature of the data, we compared the distributions of the labels used for each LS direction pairwise between the three conditions using crosstabulation and chi square tests. Clearly, multiple comparisons between a set of data could inflate a family-wise type 1 error rate, (i.e., considering a statistical difference, when in fact this result might be due to chance). The Bonferroni correction of *P-*values, which is a usual way to compensate this kind of misinterpretation, did not seem to be appropriate to our data. This is because we have a large number of multiple comparisons (16), and we are looking for many that might be significant. In such cases, the Bonferroni correction may lead to a very high rate of false negatives. Instead, we used the Benjamini-Hochberg procedure (Benjamini and Hochberg, 1995) in order to control the false discovery (positive) rate. We set our false discovery rate to 0.1, that is, we were willing to accept up to 10% of LSs with significant results being false positive. This procedure consists of the following steps: first, the individual *P-*values (given from the chi square tests, as Assymp. Sig. 2-sided) are set in increasing order. Next, each individual *P-*value is compared to its Benjamini-Hochberg critical value, (*i/m*)*Q*, where *i* is the rank (1–16), *m* is the total number of tests (16) and *Q* is the false discovery rate (0.1). Finally, identifying the largest *P-*value that has *P*<(*i/m*)*Q*, which is significant, and all of the *P-*values smaller than it are also significant, even those that are not smaller that their own Benjamini-Hochberg critical values (McDonald, 2009).

Additionally, we calculated the accuracy of head turning in Hc and HAPc, and arm pointing in HAPc, based on the spatial coordinates of the reflexive markers attached to participant’s head, arms and upper body, recorded by the Vicon system at the time of the verbal response. From these data, the coordinates of the target direction of the participant’s response movements projected onto the ring were computed and converted into degrees (in a range of 360°) using custom written Mathematica programs (Wolfram Mathematica 7). The direction of LS 0 was defined as 0° and the subsequent LSs were further graduated clock-wisely, in steps of 22.5°. For head turning movement, the coordinates in the ring refer to the projection of the vector formed by the markers at the frontal bone and above the occipital protuberance; for the arm pointing, the coordinates in the circular ring refer to the projection of the vector formed by the markers on the shoulder and index finger (see **Figure 1**).

As in earlier studies, (e.g., Philbeck et al., 2008), we analyzed the response movements in terms of signed and unsigned errors. We calculated the signed errors as the difference, in degrees, between the real direction of the LS and the response movement direction. This type of error provides indication of an overall tendency to overshoot or undershoot the location of the sound sources. Thus, errors in clockwise direction have negative sign and errors in anti-clockwise direction have positive sign. Because positive and negative signed errors can be canceled out, which might cause an underestimation of the averaged errors, we additionally analyzed the unsigned (absolute) error scores. These were calculated by averaging the differences between the response movement and the actual LS positions, ignoring the positive or negative signs. We evaluated the scores of the signed and unsigned errors of the arm pointing and head turning in HAPc and of the head turning in Hc (dependent variables) using one-way ANOVA and Sidak *post hoc* tests, with LS (0–15) as factor.

## Results

### Verbal Responses

In each response condition, Wilcoxon signed rank tests examined whether the use of the most frequent label assigned to describe each LS was significantly higher than the other labels also assigned to that LS. This reveals which are the most representative labels assigned for each LS direction. In FFc, all LSs were represented by one specific label, except LS 5, for which no difference occurred between the use of labels R and BR (for the complete descriptive statistics and results of the Wilcoxon signed rank tests in the three conditions, please see the supplementary material). In Hc and HAPc, all LSs were represented by only one concept, except LS 5 (by BR and R), LS 11 (by L and BL), and LS 13 (by FL and L).

**FIGURE 2:**
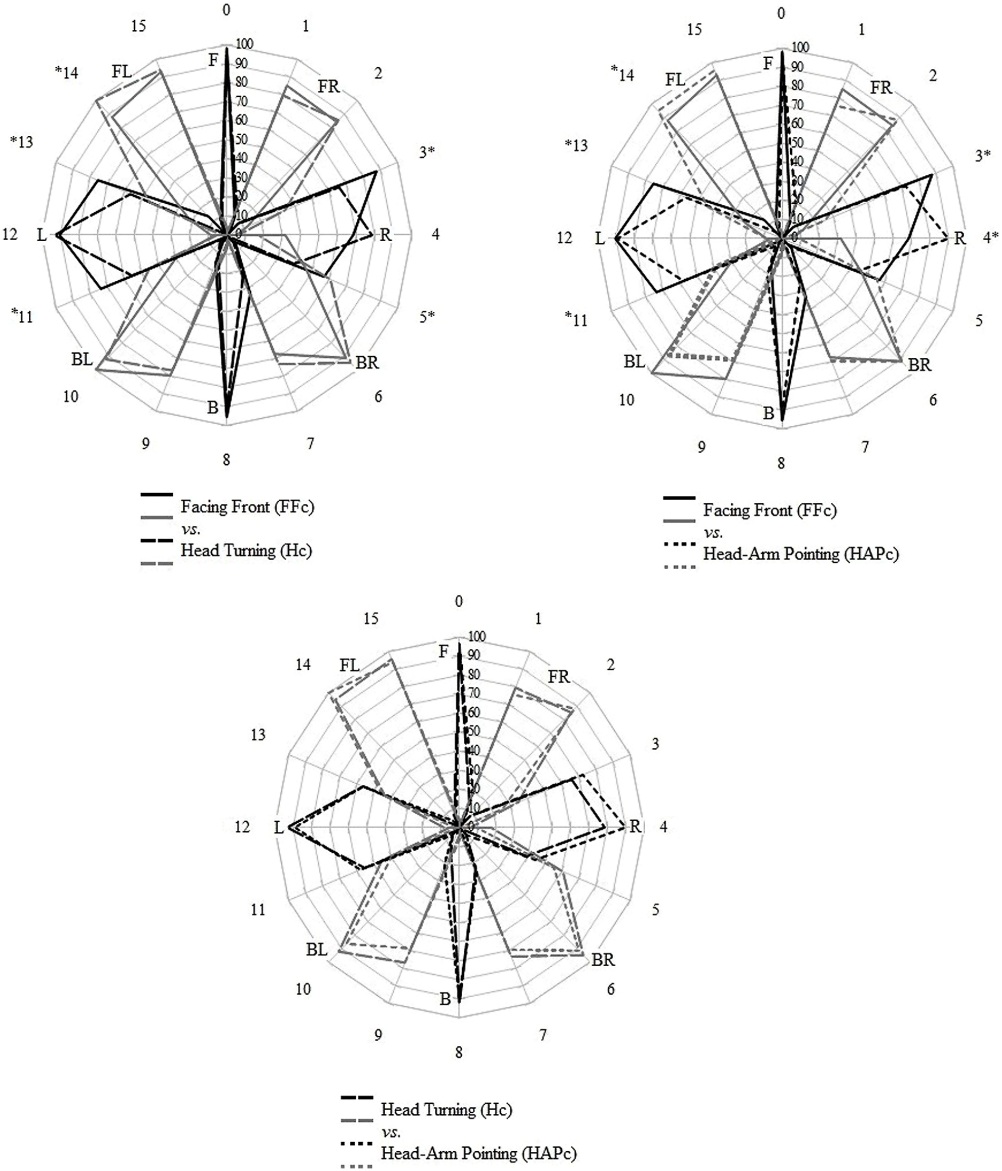
**Distribution of direction labels given in the verbal responses for each LS direction**. The numbers outside of the circles represent the LSs; the participant was facing LS 0. Black lines correspond to the simple labels (F, L, R, B) and gray lines to the combined labels (FR, FL, BR, BL). Differences between the conditions were found in the directions flagged with asterisks (Chi square tests, original *P-*value <0.05 with Benjamini-Hochberg correction). The radius of the circle indicates the maximal value of valid responses (0–100%), and the concentric lines indicate steps of 10%.

The distributions of the labels used for each LS were compared between the conditions using Chi square tests, controlling the false discovery rate with Benjamini-Hochberg procedure, and are displayed in **Figure 2**. In general, for LS directions in the front and back regions, the distributions of the verbal labels were consistent across conditions; on the sides, the distributions varied between FFc and the two other response conditions. Specifically, the labels L and R were used relatively more often for LS positions adjacent to the marginal sides (LSs 4 and 12) in FFc than in Hc and HAPc (see **Table 1** and **Figure 2**). Hc and HAPc differed only for LS 4, 6, and 12.

**Table 1:** Pearson Chi-square test for the distributions of the labels used for the loudspeaker directions between the conditions.

Conditions	Loudspeaker	Value	df	Asymp. Sig. (2-sided)
FFc and Hc	3	14.936	1	0.000
	5	6,187	1	0.013
	11	7.07	1	0.008
	13	10.756	2	0.005
	14	15,671	2	0.000
FFc and HPc	3	6.533	1	0.011
	4	17.578	2	0.000
	11	7.035	2	0.030
	13	11.563	1	0.001
	14	5.403	1	0.020

*The table displays only the directions significantly affected by the response conditions, using the Benjamini-Hochberg procedure with a false discovery rate of 0.1. The degrees of freedom are related to the number of labels used for the referred loudspeaker. We found no significant difference between Hc and HAPc*.

### Accuracy of Response Movements

**FIGURE 3:**
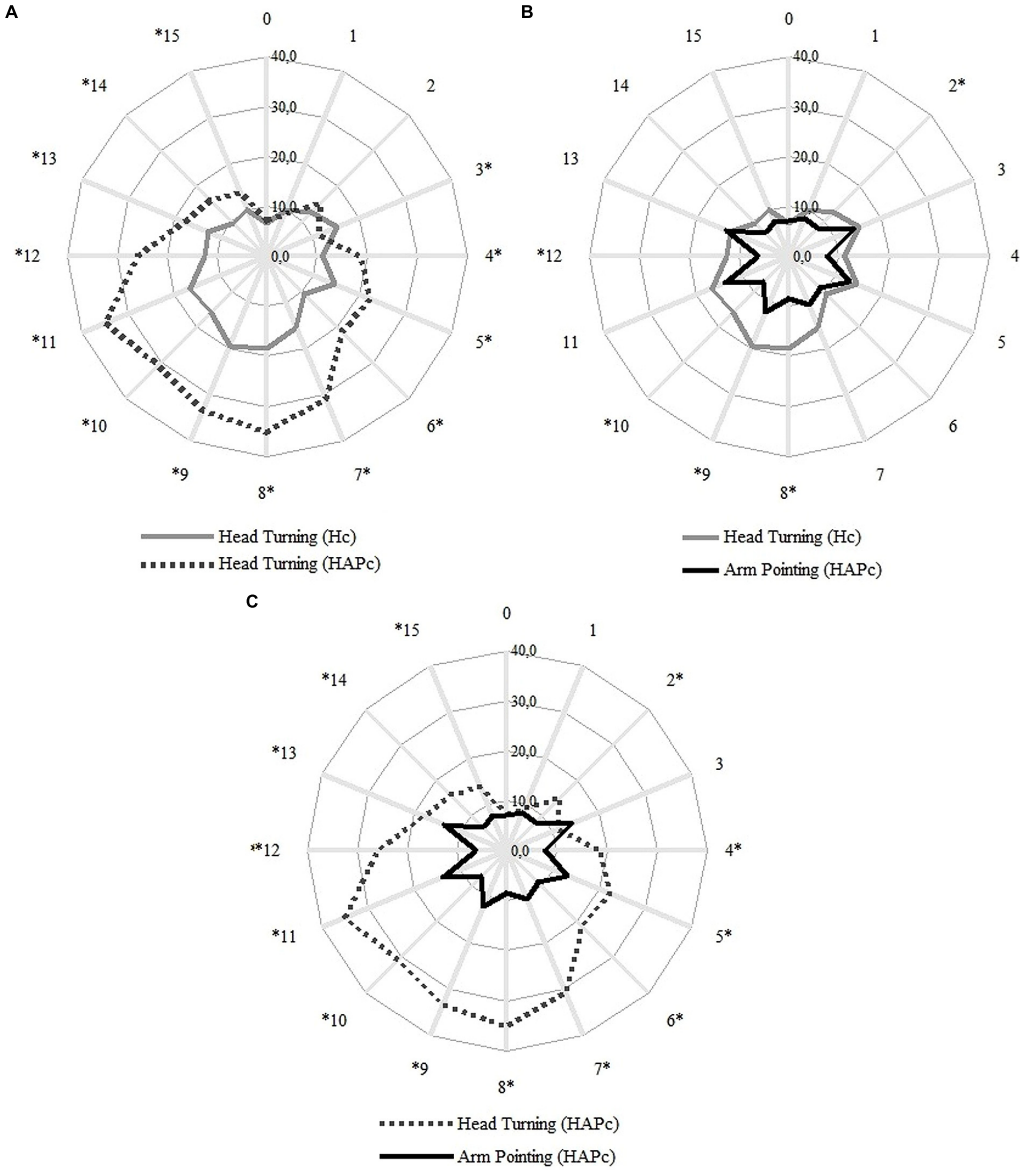
**Averaged distribution of the unsigned error for each direction in degrees**. The numbers outside of the circles represent the LSs; the participant was facing LS 0. Concentric lines indicate steps of 10° of deviation from the true LS angle. Gray full line: head turning in Hc, gray dashed line: head turning in HAPc, black full line: arm pointing in HAPc. Statistical differences (ANOVA and Sidak *Post hoc* tests, Asymp. Sig. <0.05) between head turning in Hc and HAPc **(A)**, between head in Hc and arm pointing in HAPc **(B)**, and between head and arm in HAPc **(C)**, are denoted with asterisks.

Unsigned error values of pointing varied with the response movement (head turning or arm pointing), with the response condition (Hc or HAPc), and with the direction of pointing (**Figure 3**). In Hc, head turning produced overall more errors in the rear space than in the sides and frontal spaces (**Figure 3**, gray full line). In HAPc, head turning errors followed the same pattern, but with larger errors than in the Hc (**Figure 3**, gray dashed line).

The arm pointing in HAPc generated a different pattern of errors. We found largest values of unsigned error in LSs adjacent to the canonical left, back and right, (i.e., in LSs 3, 5, 7, 9, 11, and 13), and these errors did not differ significantly from each other (ANOVA and Sidak *Post hoc* tests, Asymp. Sig. >0.05). Except for LS 7, the unsigned errors in these adjacent LSs differed from all canonical directions (i.e., from LSs 0, 4, 8, and 12; ANOVA and Sidak *Post hoc* tests, Asymp. Sig. <0.05), whereas the canonical front, right, back, and left did not differ from each other.

Most of the differences between the three response movements (head turning in Hc, head turning in HAPc, and arm pointing in HAPc) were found between head turning in Hc and HAPc, and between head turning and arm pointing in HAPc (both in 13 out of 16 LS directions). Head turning in Hc and arm pointing in HAPc were only different in LS directions 2, 8, 9, 10, and 12. The smallest errors were found in LS directions 0 and 1 in all conditions and response movements.

**FIGURE 4:**
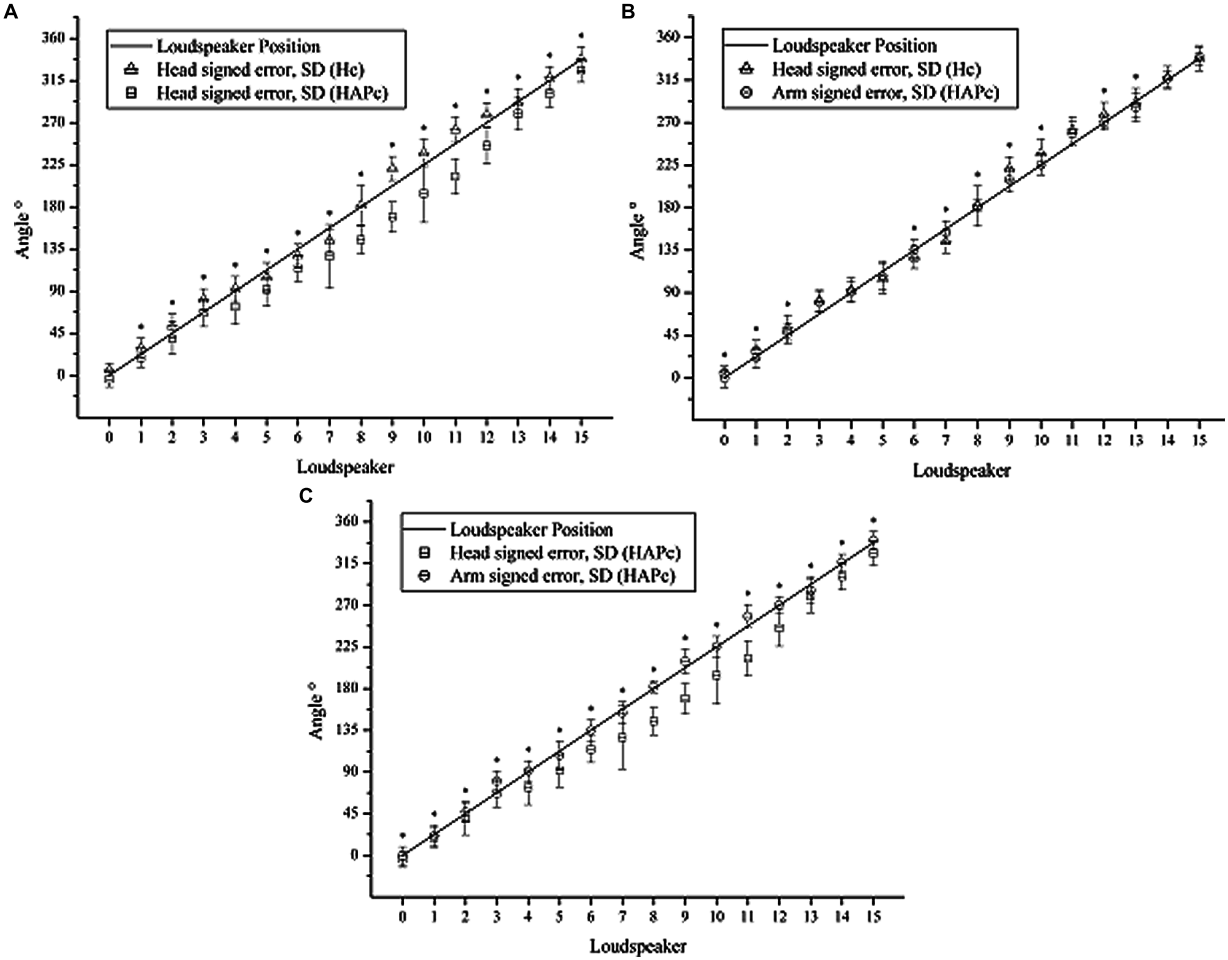
**Mean signed errors of head turning in Hc (triangles) and HAPc (squares), and arm pointing (circles)**. Line: actual angles of LS directions in relation to LS0; whiskers: standard deviation. **(A)** Head turning in Hc and HAPc; **(B)** Head turning in Hc and arm pointing in HAPc; **(C)** Head turning and arm pointing in HAPc. Asterisks indicate results of ANOVA and Sidak *Post hoc* tests at the significance levels of 0.05.

More specific than the unsigned errors, the signed errors denote the deviation (in degrees) from the original LS direction, and the sign of the averaged errors, indicating whether the LS position was generally underestimated (i.e., perceived as closer to the LS 0) or overestimated (i.e., perceived as away from LS 0). The averaged signed errors of the response movements are shown in **Table 2** and **Figure 4**.

**Table 2:** Mean response directions, mean signed error and standard deviation for the three response movements (head turning in Hc, head turning and arm pointing in HAPc).

	Head turning (Hc)		Head turning (HAPc)		Arm pointing (HAPc)	
LS position	Mean	SD	Mean	SD	Mean	SD
0	5.822	8.169	-4.252	6.301	-0.513	9.778
1	6.636	10.235	3.682	10.576	-1.255	11.212
2	7.504	17.260	5.521	13.223	2.068	10.871
3	13.669	14.928	0.553	11.379	12.494	10.834
4	2.647	18.607	16.642	12.986	1.003	11.000
5	-7.655	17.985	20.673	15.623	-4.708	15.280
6	-7.228	14.139	20.510	12.248	-0.176	11.443
7	-13.426	33.942	30.332	12.159	-3.762	12.076
8	1.717	14.769	35.148	21.145	2.021	10.349
9	18.492	16.265	-33.357	12.871	6.939	13.010
10	12.739	30.930	-30.617	14.584	0.490	10.992
11	14.952	18.824	-34.980	13.501	10.450	12.613
12	8.465	19.739	-24.631	12.958	0.522	8.366
13	-1.249	17.698	-12.778	15.073	-6.638	14.581
14	2.855	14.104	-14.314	11.844	1.255	8.774
15	0.442	12.576	-11.622	13.017	2.477	9.481

*Actual angle is the real LS position. We calculated the signed errors as the difference between the real LS position and the response movement direction. Errors in clockwise direction have negative sign and errors in anti-clockwise direction have positive sign*.

In Hc, the head turning responses tended to be shifted toward the sides (i.e., toward the rear in the frontal region, and toward the front in the rear). The same turned out for the arm pointing in the HAPc, but only for the LSs adjacent to the absolute left, right and back (i.e., 11, 13, 3, 5, 7, and 9 respectively). For these directions, the averaged directional error varied from -3.76° (LS 7) to 12.49° (LS 3), while, in the frontal region, the higher averaged error was for LS 15 (2.48°).

Pointing with the arm produced smaller bias and less variation than head turning in both conditions and thereby represented the LS positions more precisely. Head turning in Hc produced still smaller bias and less variation and thereby deviated less strongly from the LS positions than in HAPc (**Figure 4A**). In HAPc, head turning and arm pointing notably deviated from the LS positions in opposite ways for LSs 9–12 (**Figure 4C**). In general, head turning in Hc and arm pointing in HAPc differed less from each other than both differed from head turning in HAPc, which represented the LS positions the least precisely, specifically for LS positions 7–12.

## Discussion

We examined the spatial categorization of sound sources’ directions under three response conditions. Specifically, we investigated the regions associated to spatial direction labels, and the influence of the response condition on the verbal categorization of these regions. We expected that the different response conditions would induce different verbal categorization of the sides and that the most prominent regions in auditory space (front and back) would be represented in more detail and consistently categorized with the same concepts across the conditions.

As predicted by the first hypothesis, FFc produced a more generalized labeling of the directions. The more detailed categorizations of directions in Hc and HAPc are in accordance with the assertion of Boyer et al. (2013), that the auditory system efficiently processes the changes of the acoustic cues during head movements for processing motor control. In Hc and HAPc, participants were indirectly encouraged to “search” for the sound source by turning the head. These movements provide more distinctive auditory cues, allowing for a more accurate localization in comparison to the FFc, and possibly resulting in a more refined representation. Additionally, it is plausible to assume that the turning and pointing movements directly affected the perception and thereby the representation of the sound locations, in accordance with theoretical approaches based on the coupling of perception and action. The most comprehensive theoretical framework in this regard is the theory of event-coding (TEC: Hommel et al., 2001), which integrates the common coding approach (Prinz, 1990) and the action-concept model (Hommel, 1997). The common coding approach proposes a common representational domain, which is shared by perceived events and planned actions (e.g., Prinz, 1990, 1997; Gallese et al., 1999; Christensen et al., 2011). This means that not only perception affects action, but also that the planning and executing of actions influence perceptual judgments. Hommel’s (1997) action-concept model claims that human cognition is based on integrated sensorimotor units, and empirical evidence exists that concurrent motor execution can facilitate sensations in the visual (e.g., Miall et al., 2006; Christensen et al., 2011) and auditory domain (Repp and Knoblich, 2007), and that negative interference induced by actions can occur under certain conditions (e.g., Müsseler and Hommel, 1997; Hommel and Müsseler, 2006; Zwickel et al., 2010). Whether mainly grounded in the psychophysical changes of auditory cues caused by head turning, and/or in the action-based influences of head turning and pointing, the differences in spatial representation between FFc and both Hc and HAPc clearly showed that response actions lead to a more refined categorization of the auditory space.

We did not expect differences between Hc and HAPc in sound perception, because the turning movement of the head occurred in both response conditions. The second hypothesis predicted, however, a more detailed categorization in HAPc than in Hc, based on the implicit communicative function of pointing and on the proprioceptive and visual feedback of the pointing movement. Our results did not support this prediction, as we found no difference between the two response conditions. It could be assumed that the action requirements of the two conditions were not different enough to produce significant changes in the perception and representation judgments for the performed task. Notably, the task required forced choices in categorization with 12 predefined labels (which were “translated” into eight), which certainly deliver less sensitive results in comparison to tasks with broader ranges of choices, such as reporting angle or clock face descriptions (e.g., Haber et al., 1993a). Hence, we conclude that head turning and arm pointing response actions to retrieve sound locations do not lead to substantial differences in spatial perception and representation in tasks such as the ones performed in this study, but we do not exclude the possibility of finding differences in more sensitive tasks that employ these two response actions.

Confirming the third hypothesis, the effect of condition appeared specifically in the side regions, as the labels “left” and “right” were used relatively more often for LS positions adjacent to the marginal sides in FFc than in Hc and HAPc. Nevertheless, simple side labels were still frequently used to describe these regions, instead of the expected combined labels. The signed and unsigned error patterns found in this study partially explain these results. The arm pointing in HAPc and the head turning in Hc produced the largest unsigned error values in the directions adjacent to the sides, and the signed errors showed that these were biased toward the absolute left and right. Similar patterns were observed by Oldfield and Parker (1984) for auditory targets and by Franklin et al. (1995) for visual stimuli. A possible explanation is that participants named the directions adjacent to the cardinal sides with simple labels rather than combined labels, because they had indeed perceived the sounds biased to the cardinal sides. However, as the distance between the LSs was 22.5°, the localization task can be rated as rather easy, which makes such perceptual errors unlikely to occur (see Lewald et al., 2000). As an alternative explanation, we suggest that the participants’ labeling of the adjacent sound sources was influenced by the implicit importance of the side concepts. This implies a top–down influence on the conceptual level that provided a kind of “gravitational force” of the side concepts.

Interestingly, the described pattern was not observed in head turning in HAPc, the response movement that produced the largest error values. As earlier discussed, in both Hc and HAPc, the head was free to turn toward the sound’s correct location, and therefore the differences in head turning accuracy are unlikely to be based on differences in perception of the correct stimulus location in these two conditions. We assume that the discrepancy between HAPc and Hc head turning responses occurred rather because participants in HAPc did not follow the instruction of clearly facing the perceived sound source (instead, they visually “captured” the target without completing the movement of the head), as they understood the arm pointing as the implicitly more relevant response movement in this condition. If this was the case, the head turning in Hc might have obtained a communicative function additional to facilitating stimulus localization. Following this line of argument, in HAPc, in contrast, head turning only helped to localize the stimulus, whereas the arm was pointing to the perceived sound source, fulfilling a potential communicative function. This might explain why the two response conditions produced similar verbal responses despite the dissimilar head movement scores. Moreover, the fact the task required to keep the feet directed frontward throughout the experiment, certainly added an awkward constraint to the head movement, which could have affected both the perception of the correct sound location and the verbal categorization of its direction, especially for more eccentric stimuli. Indeed, the larger errors found for the head turning movements in both Hc and HAPc occurred in the rear regions, namely in the furthest directions. However, these errors do not appear to have been caused by inaccuracy in perceiving the location of the stimuli in this region, since the concomitant arm pointing movement in HAPc produced smaller errors in this region than within the sides, showing that participants were able to properly perceive the sound location. The constraints in head turning movements caused by fixing the position of the feet did not appear to affect the categorization of rear region either, since the resolution in this region was virtually as good as in the frontal region, where only small movements were required.

Notably, the differences in verbal categorization between the conditions occurred prominently in the side regions, whereas the front and back regions were categorized more consistently across the conditions. The front and back regions were distinctively defined (with simple labels used exclusively for the absolute front and back) and consistently categorized with the same concepts, whereas the labels assigned to directions adjacent to the absolute sides varied between FFc and the two other conditions. When the participant’s head was kept straight facing front (FFc), these directions were more often categorized with simple labels than with combined labels; when the head was turned toward the stimuli (Hc and HAPc), thereby facilitating its localization, the simple labels were used more specifically for the cardinal left and right, and combined labels were used instead for the adjacent directions. In this case, we assume that the response actions might have affected the representation of the auditory directions in terms of an influence from bottom–up information processing.

The consistent categorization of the front and back regions is related to the fact that directions within these regions can easily be distinguished, based on interaural time and level differences (ITD and ILD). The sign of ITD and ILD changes when crossing the front-back axis, and thus the directions to the left or to the right of this axis are very well-recognized. When crossing the left–right axis, however, ITD and ILD remain almost the same, and then the listener has to rely on monaural cues to localize the sound sources. This less clear perceptual discrimination between directions on the sides might lead to lower representational resolution within the regions that encompass these directions.

The distinctiveness of the regions in the auditory space could be additionally associated to the typical use of auditory information. To explain the reasoning for this proposition, we relate here the categorization of the auditory space found in our study with the general representation of the visual space. Studies from diverse areas have shown that spatial representation is relatively independent of a special modality of input, so that information from different senses are joined and integrated to a general spatial perception (for an extended review, see Vorwerg, 2001). In our study, although the LSs were hidden, participants could see the environment and therefore had visual feedback from the space and from their own body. This visual information might have influenced the auditory spatial cognition, providing an integrated and coordinated spatial representation. Even when sighted participants are blindfolded, they still have a visual mental map of the environment in memory, so that the relationships between the egocentric directions remain reasonably intact. Additionally, the turning movement provides proprioceptive feedback that helps the listener to perceive and categorize the directions, relative to the initial position. Due to the integrative nature of the task, it was indeed to expect that the representation of the auditory space would have commonalities with the representation of the visual space.

Auditory and visual spaces share the privileged status of the frontal region in perception and representation. As explored by Franklin et al. (1995) in the visual domain, egocentric front is more accurately perceived and represented, and more thoroughly described. The same is true for the auditory field in our study. In their study, Franklin et al. (1995) additionally found that the front region encompassed a larger area than the other regions, although the concept “front” was not used to categorize the whole extension of the region: when participants ascribed spatial concepts to the surrounding directions, front emerged as the less frequent concept used in single-direction descriptions. The authors reasoned that, when the stimulus was in the frontal region, participants tended to omit the label “front,” treating it as default, and giving responses such as “slightly to the left” when they actually meant “front, slightly to the left.” In our study, the label “front” was also used less often than the others, but the argument made by Franklin et al. (1995) cannot be applied to our participants’ behavior, as we did not allow such implicit responses. Hence, our results indicate that, in the auditory space, the front region is not only more discriminative in resolution, but also the spatial concept of front is restricted to a smaller area.

Additionally to the similar status of the frontal region in auditory and visual spaces, the representation of the back has also been shown to be specific in the human brain. For instance, Viaud-Delmon et al. (2007) provided evidence that the horizontal is separately represented in the human brain. The authors tested different visual tasks in near and far spaces in patients with left-sided neglect, and found that the patients’ failure to organize space in the left–right dimension did not affect the organization of their front-back dimension. Interestingly, they observed that only the left hemispace in front of the patient’s body was inaccessible, whereas the representation of the space behind remained intact. The authors argue that the imagery of the backspace does not share the same neural correlate as the frontal space, because in the back, it is not possible to adopt a viewer-centered reference frame, which is the basis for the imagery of the frontal space (Viaud-Delmon et al., 2007). The authors additionally discuss their results in terms of visuomotor orientation: because action planning is commonly done in the frontal space and rarely in the back, it is plausible that the former is coded with a stronger contribution from the motor system, whereas the latter involves different neural processes. As pointed out by Boyer et al. (2013), in accordance with Aytekin et al. (2008), both the acoustic inputs and their relation to motor states must be efficiently integrated for a stable representation of the auditory environment, and the role of the motor component is exacerbated in the furthest orientation, such as the back space.

Farnè and Làdavas (2002) investigated auditory and tactile integration in the peripersonal space, and observed that sounds presented in the ipsilesional space of a brain-damaged patient can induce the extinction of tactile stimuli on the patient’s contralesional side. This tactile extinction was more pronounced when the sounds occurred in the back than in the front, suggesting that information coming from the back is actively integrated in a more general representation of space, at least in the auditory domain. Using an auditory localization task, Vallar et al. (1995) observed that patients with right brain damage and unimpaired control subjects showed a greater displacement in the back space than in the front. Summarizing, these studies point toward the differential representation of the frontal and back spaces not only for the visual domain (possibly related to visuomotor orientation; Viaud-Delmon et al., 2007), but also for the auditory domain (Vallar et al., 1995), as well as an integration of the auditory back space in the spatial representation (Farnè and Làdavas, 2002). Similarly, the present study, together with the results of Campos et al. (2013), reflect the relevance of sounds occurring in the distinct surrounding regions to representation structures that appear to be specific for the auditory space, rather than merely reproducing the spatial representation acquired through vision.

The relationships between the representations of the auditory and visual spaces instigate the question in how far the availability of a visual map affects the representation of space. In a review on this regard, Kitchin et al. (1997) have identified three lines of thought, which state that the spatial representation of the blind is either deficient, inefficient, or different from the sighted. While the first and second lines refer to the lack of a visual map, the third states that any difference relative to sighted people can be attributed to intervening variables such as access to information, experience, and the amount of stimulation (e.g., Golledge, 1993; Haber et al., 1993b). The latter is in accordance with the action-specific perception account, which states that a person’s ability to interact in and with the environment influences his or her perception of this environment (e.g., Bhalla and Proffitt, 1999; Witt et al., 2005; Witt, 2011). For instance, hills were rated as steeper by people who were carrying a heavy backpack, fatigued, of low physical fitness, elderly and/or in declining health in comparison to control viewers (Bhalla and Proffitt, 1999). Softball players who were currently hitting better reported perceiving the ball as bigger than currently weaker players (Witt and Proffitt, 2005). Dart-throwing performance was demonstrated to affect the perceived size of the target (Wesp et al., 2004). Taking these and related studies into account, it is plausible to suppose that the perception of auditory events and their representation are directly related to the experience of the listener in using this information, and importantly, to the possibilities of interactions with the environment that this information affords. This again raises the issue of the specific condition of blind people, for whom the use of auditory information for orientation and locomotion is clearly more relevant than for sighted individuals. The configurations of the auditory spatial representation found in the present study provide reference for studies concerning this controversial topic in blind individuals. Investigations in this regard are currently being carried out by our group in a study with blind athletes and non-athletes, in order to explore the roles of the sight condition and level of expertise in auditory-based orientation and locomotion.

## Conclusion

Taken together, our results indicate the following: first, the response condition should be taken into account when discussing the representation of auditory space, since it is known to affect the perceptual localization of sound sources, and importantly, it affects the categorization of regions with lower resolution. Sounds coming from the sides typically evoke orientation movements, and therefore the categorization of these regions is more natural and more detailed when such movements are allowed. And second, not only the absolute front, but also the back appeared to have special status in categorization: only in these directions are the labels “front” and “back” used without additional side labels. These particularities of the auditory space representation are likely to be related to the physiological characteristics of the human auditory system, as well as to the ecological requirements of action control in the different regions.

## Conflict of Interest Statement

The authors declare that the research was conducted in the absence of any commercial or financial relationships that could be construed as a potential conflict of interest.
